# Manual Therapy (Postisometric Relaxation and Joint Mobilization) in Knee Pain and Function Experienced by Patients with Rheumatoid Arthritis: A Randomized Clinical Pilot Study

**DOI:** 10.1155/2020/1452579

**Published:** 2020-08-19

**Authors:** Mateusz Wojciech Romanowski, Maja Špiritović, Wojciech Romanowski, Anna Straburzyńska-Lupa

**Affiliations:** ^1^Department of Physiotherapy, Poznan University of Medical Sciences, Poznań, Poland; ^2^Rheumatological Centre, Śrem, Poland; ^3^Wielkopolska Physiotherpaty Centre, Poznań, Poland; ^4^Institute of Rheumatology and Clinic of Rheumatology, Charles University in Prague, Prague, Czech Republic; ^5^Department of Physical Therapy and Sports Recovery, Poznan University of Physical Education, Poznań, Poland

## Abstract

**Objectives:**

The purpose of this study was to evaluate the impact of manual therapy on the management of rheumatoid arthritis (RA) patients with knee pain.

**Materials and Methods:**

This was a small, randomized clinical pilot study. Subjects were 46 patients with diagnosed RA, randomly assigned to the manual therapy group (postisometric relaxation and joint mobilization) or control group (standard exercise). Subjects in each group had 10 sessions of interventions, once a day with one day break after the sixth day. Outcomes included the pain intensity of knee, Knee Society Score, Oxford Knee Score, and Health Assessment Questionnaire.

**Results:**

There were no statistically significant differences between groups, except for the pain intensity of the knee.

**Conclusions:**

This study suggests that manual therapy (postisometric relaxation and joint mobilization) may have clinical benefits for treating knee pain and function in rheumatoid patients. Further extended studies are expected to determine the effectiveness of manual therapy in RA patients with knee pain.

## 1. Introduction

Rheumatoid arthritis (RA) is an inflammatory, immunologically dependent, systemic connective tissue disease, leading to joint destruction and organ complications [1]. The knee is one of the most commonly affected joints in patients suffering from chronic RA [2].

The estimated prevalence is 0.5–1%. The disease presents most commonly in women with a peak incidence at 55 years. Often, one consequence is disability involving difficulties in managing daily activities, work, and leisure activities [3].

Pharmacologic treatment of RA patients commonly involves drugs such as nonsteroidal anti-inflammatories (NSAIDS), antitumor necrosis factors, disease-modifying antirheumatics, and/or corticosteroids [4]. Recent guidelines for the management of RA emphasize the use of nonpharmacologic care in addition to the use of pharmacological agents [5]. Nondrug treatment includes exercise therapy, physical modalities, orthoses and assistive devices, and self-management interventions [6].

Recent publications in the mainstream medical literature have reported the benefits of manual therapy, but there are only four studies evaluating its effects in RA patients [7–10]. More research with good-quality evidence is needed to determine the efficacy of manual therapy in the management of RA [11].

Manual therapy refers to a group of methods used by physiotherapists, osteopaths, and chiropractors, with special training [12], to improve the range of motion [13] and function [14, 15] and decrease pain [16] at the knee joint. From the point of view of terminology, the term *manual therapy* includes a lot of different techniques. In this study, we aimed to check the efficacy of postisometric relaxation technique (PIR) and joint mobilization according to our previous studies [9, 10]. PIR is one of the muscle energy techniques (METs) used to improve muscle flexibility [17, 18]. PIR leads to reduced tension within a single muscle or a group of muscles and also increased muscle tolerance to stretching, which is considered to be caused by the stimulation of the Golgi tendon organs induced by isometric contraction [19]. The proper flexibility of muscles is important in decreasing pain [20, 21]. Joint mobilization is often used to treat painful joints and typically involves the application of rhythmic oscillatory motion of the joint surfaces within the normal joint range [22]. Studies have suggested that mobilization reduces pain and improves function, while increasing the range of motion [23].

Therefore, the objective of the present pilot randomized clinical trial was to assess the impact of manual therapy on pain and knee function in patients with RA.

## 2. Materials and Methods

### 2.1. Study Design

The study was a randomized clinical pilot study with unblinded treatment and blinded outcome assessment. We followed the randomization procedure of Romanowski et al. [24]. It included eligible patients recruited from the patients who had been hospitalized in the rheumatology ward at this time with a diagnosis of RA [25], and they had pain in one knee (VAS) ≥ 4, disease activity score in 28 joints (DAS28) ≤ 5.1, and Power Doppler Ultrasonography (PDUS) ≤ grade 1 [26].

After baseline assessments, 47 patients were randomly assigned to the manual therapy group and control group. The patients were randomized to groups using two sealed opaque envelopes indicating treatment allocation. Randomization envelopes were prepared at the start of the study, and a random number sequence was obtained by flipping a coin. A research assistant not involved in the conduct of the study randomized patients, allocated treatment, and collected key data. The exclusion and inclusion criteria for this study are shown in Table 1.

All procedures performed in studies involving human participants proceeded in accordance with the ethical standards of the institutional and/or national research committee and with the 1964 Helsinki Declaration and its later amendments on comparable ethical standards. Informed consent was obtained from all individual participants included in the study.

This study was approved by the Bioethics Committee of the University of Medical Sciences in Poznan (trial number: 715/17).

### 2.2. Interventions

The manual therapy group underwent a 25-minute session of manual therapy provided by three licensed therapists with at least five years of experience who were willing to follow the study protocol and had experience in the permitted techniques. The control group underwent a 30-minute standard exercise program.

Subjects in each group had 10 sessions of interventions once a day from Monday to Saturday, a break on Sunday, and from Monday to Thursday. Drugs prescribed earlier by their specialist were used in a consistent dosage.

### 2.3. Manual Therapy Group

#### 2.3.1. Postisometric Relaxation of Muscles

Hamstrings, quadratus femoris, gastrocnemius, and the soleus: the technique begins by placing the muscle (to be treated) in a stretched position by lengthening it, to the point where the first slight resistance (or “barrier”) is felt. Next, the patient is instructed to resist this movement with minimum force, isometrically, for about 10 seconds, and then, told to relax. When the patient has indeed relaxed, a gentle release is obtained, and the muscle lengthens by “spontaneous decontraction” (relaxation) and finding a new end position (stretching) for 30 seconds. The procedure was repeated 4 times for every muscle [27].

#### 2.3.2. The Mobilization of the Patella

The following movements were made in the patellofemoral joint: transverse (medial and lateral), longitudinal (caudad and cephalad), and in the direction of axial rotation [22] for 2 minutes in total.

#### 2.3.3. The Mobilization of the Knee Joint

Tibia posterior glide [28]: the patient is positioned in a supine posture with the knee slightly flexed and a prop placed under the distal femur. The stabilizing hand is used to prop the distal femur, and the mobilizing hand is placed over the proximal tibia just below the tibial tuberosity. The mobilization itself was performed by a force perpendicular to the line of the tibia for 2 minutes in total. Tibia anterior glide [28]: the patient is prone. The knee is flexed 90 degrees with the patient's feet leaning on the therapist's shoulder. The therapist's hands are on the proximal part of the tibia. The therapist stabilizes the extremity with the shoulder and pulls the proximal part of the shin with the hands. Mobilization was performed for 2 minutes in total.

Safety in this study was addressed by doing mobilization and postisometric relaxation without pain and adverse event during and after treatment. In the case of pain or any other adverse event, an adverse event form would be filled. Manual therapists were obligated to ask the patient right after treatment if the treatment was manageable for them. Every next treatment was provided by the therapist when the patient reported no increase in pain and no adverse events. Physicians were on call during whole stay of participants in hospital.

For the postisometric relaxation intervention, the subject had passively stretched muscle until a point of mild discomfort, without pain reported. For the mobilization intervention, only gentle low-velocity low-amplitude mobilization was performed which could not be painful for the patient.

Joint mobilization safety was of primary concern of this study, so only grade I to II manual mobilization of joints was performed. Such mobilization provides sufficient traction movements to counteract compressive forces in the joint while avoiding soft tissue stretching [22].

### 2.4. Control Group

In the control group, a standard exercise program, consisting of five exercises, was conducted. Cycle ergometry for 8 minutes [29] (10-11 on the Borg rating of the perceived scale). Isometric quadriceps exercise with the patients lying in a supine position: a rolled-up towel was placed beneath the knee. They were instructed to maximally activate their thigh muscles in order to straighten their knee and hold the contraction for five seconds. Isometric hip adduction exercise with the patients lying in a supine position: a small cushion was placed between the knees. They were instructed to perform isometric hip adduction exercise while pressing the pillow between the knees and to maintain the adduction with a contraction for five seconds [30]. Isotonic quadriceps contraction was held in midflexion for five seconds (the subject sits in a chair, lifts the lower leg to a partially extended position, and holds). Then, isotonic hamstring contraction (five seconds) was carried out by knee flexion (the subjects lies prone or on the side and bends the knee bringing the foot towards body) [31]. The exercises were initiated in the abovementioned order and increased to a maximum of 15 repetitions for each leg.

## 3. Outcome Measures

Subjects were assessed before treatment and one day after the treatment.

### 3.1. Primary

The Health Assessment Questionnaire (HAQ) is a disease-specific instrument that measures disability with scores ranging from 0 to 3. The instrument has demonstrated satisfactory reliability, validity, and sensitivity to change in studies for patients with RA [32].

### 3.2. Secondary

Pain intensity was assessed on a Visual Analog Scale (VAS) [10]. Participants were asked to place a line perpendicular to the VAS line at the point that represents their lower back pain during the past week anchored by “no pain” (score of 0) and “most severe pain” (score of 100).

The Oxford Knee Score (OKS) [33] is a questionnaire consisting of 12 questions relating to the knee, which the patient answered on a scale of 0 to 60 points. These questions focused on the appearance of knee pain during various activities. Each of the study participants was obliged to give one answer to each question. The number of points obtained helped to determine the incidence and severity of the inflammation. The higher the score, the lower the probability of occurrence of inflammation in the knee.

The Knee Society Score (KSS) [34] is a scoring system used to rate the knee (intensity and frequency of pain and the range of motion) and the patient's functional abilities such as walking and stair climbing. The higher the number of points obtained, the more efficient the functionality of the knee joint.

### 3.3. Statistical Analyses

Quantitative data were presented by the mean and standard deviation (SD). All quantitative results were first verified by a normality test (Shapiro–Wilk test). For comparison between manual therapy and control groups for baseline data, the unpaired-t test was used. To check whether the interaction between groups and interventions affect the outcomes in time, the repeated measure ANOVA was used. A proper Bonferroni test was performed for multiple comparisons after achieving a significant result in the main ANOVA. The *χ*2 test was used for comparing proportional data.

Statistical analyses were performed using statistical analysis software Statistica PL 13.0 (StatSoft, Inc, Tulsa, OK, USA). A *p* < 0.05 was considered statistically significant.

## 4. Results

**Figure 1:**
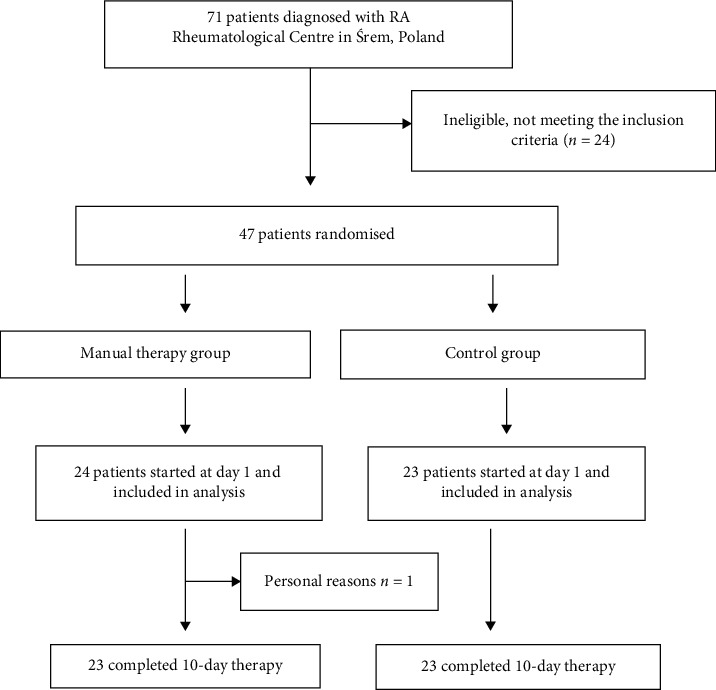
Trial profile.

The flow of the participants through the trial is shown in Figure 1. Based on the exclusion and inclusion criteria, 47 patients out of 71 were included in this trial. For personal reasons, 1 more patient was excluded from this study.

Patients from the manual therapy group and control group did not differ in basic characteristics before therapy in terms of age, body mass, height, DAS28, or BMI (Table 2).

### 4.1. Primary Outcome Measure

**Figure 2:**
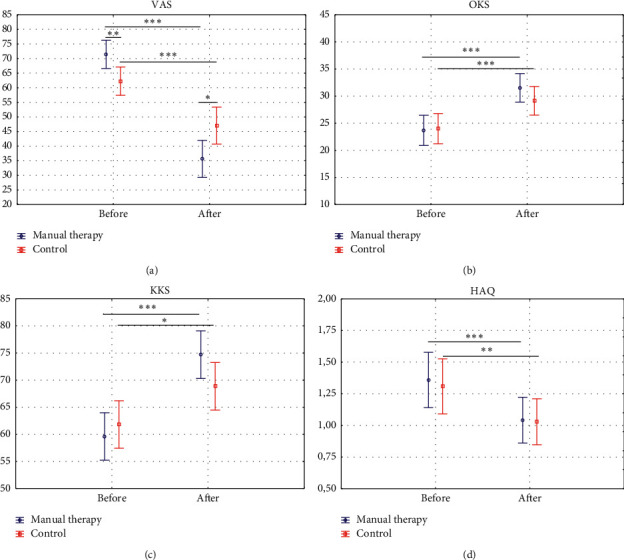
Mean score differences over time according to group allocation using intention to treat analysis. (a) VAS. (b) OKS. (c) KKS. and (d) HAQ.

Patients from the manual therapy group and the control group did not differ in the Health Assessment Questionnaire before the start of therapy. After therapy, there were no significant differences between groups (Figure 2, Table 3).

### 4.2. Secondary Outcome Measures

Patients from the manual therapy group and control group did not differ before therapy in the Knee Society Score and Oxford Knee Score, except in the VAS.

After intervention, the manual therapy group showed a significantly greater reduction in the VAS than the control group. There were no signiﬁcant differences in the Knee Society Score and Oxford Knee Score between the groups (Figure 2, Table 3).

## 5. Discussion

This was a small, randomized clinical pilot study to investigate the effectiveness of manual therapy techniques: postisometric relaxation and low-grade joint mobilization on the severity of knee pain and function in RA patients. This study is, to our knowledge, the first research of manual therapy of the knee in RA. Manual therapy was carried out in accordance with the proposed protocol and safety principles, and no adverse events were reported. So far, a well-documented form of treatment in an RA patient is physical exercises, and reliable research in the field of manual therapy is missing [11]. But, there is “professional agreement” for passive mobilizations to maintain or restore the range of motion [35].

The main finding of this study was that manual therapy (postisometric relaxation and joint mobilization) significantly decreased pain and that improved mobility of the knee joint in patients with RA. However, there were no significant differences between the manual therapy group and the control group, except for pain.

It is very important that manual therapy techniques are always matched to the individual needs of the patient. For patient diagnosis, disease activity, palpation and visual assessment, functional tests, range of joints motion, joint play, muscle and fascia mobility manual techniques should all be addressed. The manual therapists should pay attention to how the tissue reacts to the particular technique and continually analyze it until it starts loosening up, so manual therapy is individually matched to the patient, especially in RA patients who might have synovial tissue proliferation, persistent inﬂammation, cartilage degradation, bone erosion, and damage to the adjacent soft tissue and neural structures [36]. Manual therapy should be effective but above all safe. Of utmost importance are the palpation skills of the therapist, which help to locate strained muscle fibers and trigger points and assess joint play, ligaments, and fascia.

Manual therapy may alter the imbalance between facilitatory and inhibitory inputs, thereby enhancing descending pain modulation [37]. Joint mobilization has been reported to have some effect on pain in patients with knee osteoarthritis [38]. Manual therapy improves function and maximizes the circulation of nutrients to the joints and the removal of waste [39]. The enhanced hypoalgesic effect of repetitive mobilization may reﬂect changes in the local cellular environment [38]. Muscle energy techniques, of which postisometric relaxation is the most frequently applied, are effective in improving reported pain, disability, and joint range of motion in both asymptomatic subjects and symptomatic patients [40]. However, the mechanisms behind the clinical effectiveness of MT have not been established [41].

We found only four articles in the current literature where manual therapy was used to treat an RA patient. Levitsky et al. [8] suggest that manual therapy consisting of low-grade mobilization of metacarpophalangeal (MCP) appears feasible, safe, and effective for patients with RA. Despite most participants having no Doppler signal in the MCP joints (grade 0-1) at baseline, there were significant reductions in pain and increases of MCP joint space over four weeks of treatment and subsequent one-month follow-up. The randomized hand was treated for 28 minutes per session, twice within two weeks, and then, the randomized treated hand was crossed over to control (untreated) during weeks three to four and vice versa. This study was the first randomized trial in treating the hands of RA patients with manual mobilization. In the next study, 10 days of PIR on three groups of muscles, knee flexors, knee extensors, and plantar flexors, of the foot showed statistically significantly decreases in knee pain in patients with RA (*p* ≤ 0.001). PIR was statistically significantly better at decreasing pain than kinesiotherapy (*p*=0.0147) [10]. Two case studies involving manual therapy in RA patients were found. Chung [7] reported relief in pain after manipulation in one patient with acute symptomatic thoracic spine pain and RA. Due to the possible fragility of anatomical structures in patients with RA, more additional scientific research in the manipulation technique area is warranted before using them in an RA patient. In the fourth study, 10 days of mobilization (radiocarpal joint, metacarpophalangeal joints, and interphalangeal joints), each session totaling 22 minutes, showed decreased pain and improved dexterity and grip strength of the hand of a patient with RA [9].

In conclusion, this study is the first randomized clinical pilot study to investigate the effectiveness of manual therapy (postisometric relaxation and joint mobilization) on the severity of knee pain and function in RA patients. This study suggests that treatment based on manual therapy, postisometric relaxation and joint mobilization, could be considered to supplement the comprehensive treatment of an RA patient. Due to the small group sizes and lack of follow-up data, the results obtained do not lead to any final conclusions about the role of manual therapy in the treatment of RA, but are very promising and may prompt further studies on using manual therapy in RA patients.

### 5.1. Study Limitations

The present study was a single-blind study; it was not possible to blind either patients or therapists for the allocated treatment performed in groups. Therefore, extra attention was given to the blinding of the outcome assessor. We cannot exclude the possibility of a placebo effect due to the nature of the interventions. Furthermore, another limitation of the study may be the relatively small number of patients who received therapy during the research. After intervention, the manual therapy group showed a significantly greater reduction in VAS than the control group; however, the groups did differ in the VAS before the start of therapy. It should be noted that, to minimize the potential negative effect of pharmacotherapy on the researched parameters, no changes in treatment before the start and for the duration of the study were performed. The study did not provide any long-term follow-up.

The primary outcome measure was the functional status, assessed with the Health Assessment Questionnaire, with well-established validity in RA. It was selected because of its ability to measure patient activities of daily living, and daily activities can be dependent on function and pain of the knee joint. However, the short-term assessment of the outcome is a limitation of this study, as it may take time for functional health to improve. In order to demonstrate the possible impact of manual therapy on the Health Assessment Questionnaire, the next study may be improved with long-term treatment and follow-up.

## Figures and Tables

**Table 1:** Criteria for inclusion and exclusion.

Inclusion in the study	Exclusion from the study
Diagnosis of RA	Change in dosage of nonsteroidal anti-inflammatory drugs and corticosteroids for 2 weeks before the beginning of the study and for the duration of the study
Age range: participants had to be of 18 years or above	Change in dosage of the disease-modifying synthetic and biologics antirheumatic drugs for 3 months before the beginning of the study and for the duration of the study
Experience daily pain of one knee (VAS ≥4) with power Doppler ultrasonography ≤ grade 1	Injection of a local anaesthetic and steroids for 1 month before the beginning of the study and for the duration of the study
DAS28 ≤ 5.1	Every recent surgery in the past 6 months
Informed consent for participation in the study, signed by the patient	History of knee joint replacement in the treated knee
	Neurological signs present
	Experience daily pain of both knees
	Chronic bone damage
	Soft tissue injuries in the knee
	Acute inflammation within the latest week in any joint of lower limb joints
	Pregnancy
	Fever

**Table 2:** Baseline characteristics of the 46 patients with knee pain and with rheumatoid arthritis.

	Manual therapy group (*n* = 23)	Control group (*n* = 23)	*t*	*p*
Age (years)	59.7 (12.15)	57.7 (8.00)	0.66	0.5131
Body mass (kg)	75.9 (12.20)	70.3 (16.20)	1.33	0.1917
Body height (cm)	159.4 (8.30)	160.0 (5.87)	−0.33	0.7444
DAS28	4.66 (0.43)	4.31 (0.90)	1.12	0.2289
BMI (kg/m^2^)	30.1 (5.49)	27.5 (6.61)	1.41	0.1649
NSAID (*n*)	16	17		
DMARDsynthetic (*n*)	11	13	*χ * ^2^ = 0.22	0.8972
DMARDbiologics (*n*)	7	6		

The results are expressed as mean (SD); BMI: body mass index; DAS 28: disease activity score; VAS: visual analog scale; NSAID: nonsteroidal anti-inflamatory drugs; DMARDs: disease-modifying antirheumatic drugs; *p*: *p* values.

**Table 3:** Mean score differences over time according to group allocation using intention to treat analysis.

	Manual therapy group		Control group		ANOVA		
	Before	After	Before	After	*p*		
					Group	Time	Interaction
VAS	71.43 (12.52)	35.65 (12.15)	62.26 (10.50)	47.04 (17.52)	0.739	<0.001	<0.001
OKS	23.70 (7.29)	31.50 (26.78)	24.00 (5.82)	29.10 (35.71)	0.559	<0.001	0.052
KKS	59.61 (12.94)	74.70 (10.15)	61.80 (37.08)	68.87 (10.71)	0.518	<0.001	0.004
HAQ	1.30 (60.57)	1.00 (40.44)	1.30 (10.46)	1.00 (30.42)	0.811	<0.001	0.696

The results are expressed as mean and standard deviation (SD); VAS: visual analog scale; KSS: Knee Society Score; OKS: Oxford Knee Score; HAQ: Health Assessment Questionnaire; *p*: *p* values ^*∗*^^*∗*^^*∗*^*p* < 0.001; ^*∗*^^*∗*^*p* < 0.01; ^*∗*^*p* < 0.05.

## Data Availability

The outcome measures data used to support the findings of this study are available from the corresponding author upon request
